# Supplementary material for: Patterns of vascular access among chronic kidney disease patients on maintenance hemodialysis at Muhimbili National Hospital. A single centre cross-sectional study
Source: PLOS Glob Public Health. 2024 Nov 20;4(11):e0003678. doi: 10.1371/journal.pgph.0003678 (PMC11578468; doi:10.1371/journal.pgph.0003678)
Supplement: S1 Checklist — (DOC) [file pgph.0003678.s001.doc]

STROBE Statement—Checklist of items that should be included in reports of ***cross-sectional studies***

|  | Item No | Recommendation |
| --- | --- | --- |
| **Title and abstract** | 1 | (*a*) Page 2 |
|  |  | (*b*) Page 2 |
| Introduction | | |
| Background/rationale | 2 | Page 3 |
| Objectives | 3 | Page 3 |
| Methods | | |
| Study design | 4 | Page 4 |
| Setting | 5 | Page 4 |
| Participants | 6 | Page 4 |
| Variables | 7 | Page 4 |
| Data sources/ measurement | 8* | Page 4 |
| Bias | 9 | Page 4 |
| Study size | 10 | Page 4 |
| Quantitative variables | 11 | Page 4 |
| Statistical methods | 12 | (*a*) Page 4 |
|  |  | (*b*) NA |
|  |  | (*c*) NA |
|  |  | (*d*) NA |
|  |  | (*e*) NA |
| Results | | |
| Participants | 13* | (a) Page 6 |
| Descriptive data | 14* | (a) Page 6 to 8 |
|  |  | (b) Indicate number of participants with missing data for each variable of interest |
| Outcome data | 15* | Page 7 and 8 |
| Main results | 16 | Page 6 to 8 |
| Other analyses | 17 | NA |
| Discussion | | |
| Key results | 18 | Page 9 and 10 |
| Limitations | 19 | Page 11 |
| Interpretation | 20 | Page 10 and 11 |
| Generalisability | 21 | Page 10 and 11 |
| Other information | | |
| Funding | 22 | Page 11 |

*Give information separately for exposed and unexposed groups.

**Note:** An Explanation and Elaboration article discusses each checklist item and gives methodological background and published examples of transparent reporting. The STROBE checklist is best used in conjunction with this article (freely available on the Web sites of PLoS Medicine at http://www.plosmedicine.org/, Annals of Internal Medicine at http://www.annals.org/, and Epidemiology at http://www.epidem.com/). Information on the STROBE Initiative is available at www.strobe-statement.org.
