# Supplementary material for: Patterns of vascular access among chronic kidney disease patients on maintenance hemodialysis at Muhimbili National Hospital. A single centre cross-sectional study
Source: PLOS Glob Public Health. 2024 Nov 20;4(11):e0003678. doi: 10.1371/journal.pgph.0003678 (PMC11578468; doi:10.1371/journal.pgph.0003678)
Supplement: Attachment — Submitted filename: Responce to Reviewers.pdf [file pgph.0003678.s003.pdf]

Subject: Rebuttal Letter for Manuscript – "Patterns of Vascular Access Among Chronic Kidney Disease Patients on Maintenance Hemodialysis at Muhimbili National Hospital: A Single-Centre Cross-Sectional Study"

Dear Editor,

Thank you for the opportunity to revise our manuscript. We have made the following revisions: clarified the methodology, expanded the discussion of study limitations, and refined the conclusion for conciseness as requested. A detailed response addressing all points is attached.

We hope these revisions meet the journal's standards and look forward to your feedback

Sincerely

Daniel Msilanga

Reviewer 1		
**No;**	**Comment**	**Response**
1	Are there public renal registries in Tanzania? Is it possible to estimate the number of patients in hemodialysis per million inhabitants and/or the number of patients living with CKD in the country?	There is currently no renal registry in Tanzania; however, we are actively working to establish one. We have added the estimated number of patients on hemodialysis per million inhabitants as documented in the publication by Furia in Kidney360 to the introduction.
2	Can the authors provide further information concerning the number of dialysis centers, number of patients on hemodialysis, number of nephrologists and of vascular surgeons currently available in Tanzania?	We have included details regarding the number of dialysis centers, patients on hemodialysis, nephrologists, and vascular surgeons in the last paragraph of the introduction, as cited in Furia's publication..
3	Can the authors elaborate on how the data collected at Muhimbili National Hospital could be further extrapolated to provide a broader understanding of the Tanzanian picture at predialysis/dialysis care?	We appreciate your inquiry. While our study focuses on data from Muhimbili National Hospital (MNH), a tertiary center, we believe it provides a vital snapshot of vascular access care in Tanzania, which we have elaborated on in the discussion section
4	Can the survival rate of patients in Muhimbili National Hospital be compared to other centers where best practices in the transition to hemodialysis initiation and vascular access maintenance are in place?	Thank you for your question. Unfortunately, we do not have survival rate data from MNH. Our study did not track long-term outcomes, limiting direct comparisons with centers that employ best practices. We are in the process of establishing a dialysis registry to improve tracking of patient care
5	Are there any differences between the public and private sectors concerning predialysis care and vascular access management in Tanzania? Would it be possible to compare results from these sectors (e.g. mortality, prevalence of AVF, etc)?	Thank you for the comment. There is no difference in the initial access but majority of patients using insurance transition to either AVF or tunneled catheter while those with insurance, majority remain with non-tunneled catheter. We don't have mortality data but added data on changing access and insurance in the results section.

6	Considering the data presented in the manuscript to be a picture of the current practices in Tanzania, can the authors discuss in more detail what immediate measures could be put in place to change the ongoing practices in the short, middle and long terms? (e.g broad education programs, improved infrastructure, patient advocacy, universal public care, budget relocation, etc).	Thank you for the comment, this has been added in the discussion section.
7	Please check the spelling at line 92: (Data analysis: “escriptive” statistics were used)	The spelling error has been corrected.
**Reviewer 2**		
**No**	**Comment**	**Response**
1	A native speaker should check the manuscript and if possible an expert may help to revise their manuscript	Thank you for your feedback. We recognize the importance of a native speaker's review and have submitted the manuscript to an expert in nephrology for further revision
2	Although they only give descriptive information, Might be better to mention in more detail about analysis method.	We will provide additional details on our analysis methods
3	The sample size is small but the findings are very interesting and very important, (%95!!!) almost all patients started dialysis with non tunnelled catheter (26% had femoral catheter). uninsured rate is very high!!! these patients had only 1 session/week.-Unfortunately, the sample size is very small and may not reflect the whole country	Thank you for your thoughtful feedback. I agree that the sample size is a limitation of our study. However, since Muhimbili National Hospital (MNH) is one of the zonal hospitals in Tanzania, we believe that the practices observed here are likely reflective of similar settings (public facilities) across the country. It is important to note that we did not include private hospitals, where practices may differ, which is another limitation to consider. Despite the small sample size, the findings regarding the high rate of non-tunneled catheters and the significant uninsured rate highlight critical issues in renal care that warrant further investigation
4	I recommend the to add more centers to enlarge sample size, this will make your findings more meaningful	Thank you for your recommendation. We acknowledge that adding more centers could enhance the sample size and the overall significance of our

		findings. However, due to logistical constraints, we were unable to include additional centers at this time. We have noted this limitation in the manuscript to provide context for our results.
5	The presentation of data is poor	Thank you for your feedback regarding the data presentation. We recognize that clear presentation is essential for understanding our findings. We have made efforts to review and improve our results section, but if there are specific areas you believe need further attention, we would greatly appreciate your guidance. Your insights will help us enhance the clarity and impact of our manuscript
6	it might be interesting to compare the outcome (infection, replacement, hospitalization, mortality etc.) of patients with different vascular access types. This will help to make your findings more interesting	Thank you for your suggestion regarding comparing outcomes such as infection rates, replacement, hospitalization, and mortality among patients with different vascular access types. We acknowledge that this comparison could provide valuable insights and enhance the significance of our findings. However, due to the limitations of our study, including the small sample size and the lack of comprehensive data on these outcomes, we were unable to conduct such analyses.
**Reviewer 3**		
**No;**	**Comment**	**Response**
1	Good cross-sectional study to identify challenges of vascular access in a LMIC public dialysis Centre. Maybe the authors could give recommendations on how to improve the Fistula first initiative , patients to start dialysis with AV fistula instead of a temporary line	Thank you for your positive feedback. We have included recommendations for improving the Fistula First initiative in the conclusion section
